# Visual, vestibular, and ocular motor changes during nitroglycerin‐triggered vestibular migraine

**DOI:** 10.1111/head.70194

**Published:** 2026-09-05

**Authors:** Maria Dolores Villar‐Martinez, Francesca Puledda, Caroline Domingos‐Belo, Adolfo M. Bronstein, Peter J. Goadsby

**Affiliations:** ^1^ NIHR King's Clinical Research Facility, SLaM Biomedical Research Centre and Wolfson Sensory Pain and Regeneration, Institute of Psychiatry, Psychology and Neuroscience King's College London London UK; ^2^ Department of Brain Sciences Imperial College London UK; ^3^ University College London Hospitals (NHNN and RNTNE) London UK; ^4^ Division of Biomedical Sciences King Abdullah University of Science and Technology Thuwal Saudi Arabia

**Keywords:** Alice in Wonderland syndrome, aura, nitroglycerin provocation, nystagmus, vestibular migraine, visual symptoms

## Abstract

**Background:**

Intravenous nitroglycerin (NTG) is a well‐established model for provoking migraine attacks, but it has not previously been used to characterize vestibular migraine (VM). Different types of nystagmus have been reported during spontaneous VM attacks. We aimed to measure changes in nystagmus during NTG‐induced migraine in participants with VM.

**Methods:**

We studied 20 participants with definite VM (Barany–International Classification of Headache Disorders, 3rd edition criteria) undergoing NTG provocation in a prospective, within‐subject repeated‐measures pharmacological provocation study under three conditions at the National Institute for Health and Care Research King's Clinical Research Facility, London, between November 2024 to December 2025. Median (interquartile range [IQR]) age was 32 (29 to 38) years, and 16/20 were female. 3D video‐oculography was used to quantify total slow‐phase velocity (SPV) (°/s) of nystagmus without fixation in the center, left, right, up, and down positions. Recordings were repeated before NTG, after NTG, and after subcutaneous sumatriptan. Subjective vestibular symptoms and headache intensity were quantified with an ordinal numeric scale. Longitudinal changes in SPV were analyzed.

**Results:**

All the participants developed headache, with median peak severity 6/10 (IQR 4 to 7). Vestibular symptoms were triggered in 14/20 participants following NTG, with a median maximum intensity of 2.5 (IQR 0 to 4). Median (IQR) summed SPV (°/s) was 2 (1 to 3.25) at baseline, 1.5 (0 to 4.75) following NTG, and 1 (0 to 2.75) after sumatriptan. In Tweedie generalized linear mixed‐effects models, no significant group‐level effect of condition on total SPV was detected. Estimated marginal means were 1.87 (95% confidence interval [CI] 1.02 to 3.41) at baseline, 2.10 (95% CI 1.16 to 3.80) following NTG, and 1.73 (95% CI 0.93 to 3.19) after sumatriptan. Compared with baseline, neither NTG (*β* = 0.118, standard error [SE] = 0.267, *P* = 0.658) nor sumatriptan (*β* = −0.078, SE = 0.278, *P* = 0.780) showed significant effects. NTG provocation was associated with complex perceptions such as visual symptoms (10/20) and Alice in Wonderland syndrome type or dissociative symptoms (6/20), including derealization/depersonalization, metamorphopsia, macropsia and pelopsia, dysmorphopsia, and distorted passage of time.

**Conclusions:**

Quantitative slow phase velocity measures of nystagmus did not show a uniform group‐level change during NTG provocation in VM, despite frequent subjective vestibular symptoms. VM episodes may reflect altered central sensory processing and vestibular hypersensitivity rather than changes in vestibulo‐oculomotor output.

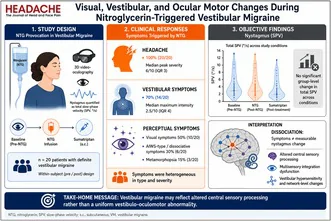

## Plain Language Summary

Vestibular migraine (VM) is a common cause of dizziness, but little is known about how the balance and visual systems interact during an attack. We injected nitroglycerin to 20 people with vestibular migraine to trigger migraine symptoms and recorded their eye movements before and after nitroglycerin and after treatment with sumatriptan. All participants developed headache, and 14 experienced vestibular symptoms. Some also reported visual distortions or feelings of unreality. However, nitroglycerin did not produce consistent changes in eye movements across the group. These findings suggest that VM symptoms may result from altered processing of sensory information in the brain rather than from consistent abnormalities in eye movement control.

## Plain Language Summary

Vestibular migraine (VM) is a common cause of intermittent dizziness, but there have been no studies with provoked (induced) VM used to characterize how the vestibular and ocular (vision) systems interact. We injected 20 patients with VM with nitroglycerin to provoke an attack and used a specialized camera to record their eye movements; we found that nitroglycerin reliably provoked VM attacks but did not find any consistent changes in eye movement. These findings suggest that VM reflects altered processing of sensory inputs in the brain and heightened sensitivity to vestibular inputs (sensory signals from the inner ear).

**Abbreviations**
5‐HT1B/1D: 5‐hydroxytryptamine 1B/1D receptor
AIWS: Alice in Wonderland syndrome
CI: confidence interval
D1: Dizziness type 1
D2: Dizziness type 2
DHI: Dizziness Handicap Inventory
ECG: electrocardiogram
ELISA: enzyme‐linked immunosorbent assay
ENT: ear, nose, and throat
F: female
IBS: irritable bowel syndrome
ICHD‐3: International Classification of Headache Disorders, 3rd edition
IQR: interquartile range
IR: infrared
L: left
LED: light‐emitting diode
M: male
MIDAS: Migraine Disability Assessment
MRI: magnetic resonance imaging
NHNN: National Hospital for Neurology and Neurosurgery
NHS: National Health Service
NIHR: National Institute for Health and Care Research
NTG: nitroglycerin
OKN: optokinetic nystagmus
PPPD: persistent postural‐perceptual dizziness
PTSD: post‐traumatic stress disorder
R: right
REC: Research Ethics Committee
RNTNE: Royal National Throat, Nose and Ear Hospital
SD: standard deviation
SE: standard error
SPSS: Statistical Package for the Social Sciences
SPV: slow‐phase velocity
UCLH: University College London Hospitals
UHD: ultra‐high definition
UK: United Kingdom
UNK: unknown
VM: vestibular migraine
VNG: video‐nystagmography
VSS: visual snow syndrome

## INTRODUCTION

Vestibular migraine (VM) is a disorder in which individuals with migraine experience recurrent vertigo and altered perceptions of space and motion. Although the association between migraine and episodic vertigo has long been recognized, formal diagnostic criteria from the Bárány Society and the International Classification of Headache Disorders, 3rd edition (ICHD‐3), have only recently provided a structured framework for classification.^1^, ^2^, ^3^

Despite consensus criteria, VM remains underrecognized in routine practice and can overlap with other vestibular disorders, contributing to diagnostic ambiguity and mechanistic uncertainty.^4^ The biological underpinnings of VM remain incompletely defined.

Spontaneous and positional nystagmus have been described during acute VM episodes; however, findings are heterogeneous and do not define a reproducible vestibulo‐oculomotor signature.^5^ This variability has generated debate regarding whether VM reflects peripheral vestibular dysfunction, transient brainstem involvement, or altered multisensory integration within migraine‐related neural networks.^6^

Intravenous nitroglycerin (NTG) is widely used in migraine research because it reliably triggers delayed attacks in susceptible individuals under controlled experimental conditions, facilitating mechanistic investigation;^7^, ^8^ however, the application of NTG provocation to VM has not been evaluated, particularly with respect to quantitative oculomotor behavior.

Applying NTG provocation in VM offers an opportunity to determine whether experimentally induced attacks are accompanied by measurable changes in nystagmus, and whether vestibular symptom generation corresponds to reproducible alterations in vestibulo‐oculomotor output. Clarifying this relationship may help distinguish between peripheral, brainstem, and higher‐order network mechanisms underlying vestibular symptoms in migraine.

The primary objectives of this study were to determine whether NTG can reliably trigger VM attacks in individuals with the condition, and to characterize the clinical and perceptual phenotype of NTG‐induced attacks. Secondary objectives included examination of whether NTG provokes measurable changes in eye movements in VM. We hypothesized that NTG provocation would produce measurable changes in nystagmus slow‐phase velocity (SPV) in participants with VM, and that these changes would be attenuated following subcutaneous sumatriptan.

## METHODS

### Ethics approval

The study was approved by the Westminster Ethics Committee (REC 23/PR/1402). All procedures were conducted in accordance with the Declaration of Helsinki and local research governance frameworks. All participants provided written informed consent.

Prior to ethics submission, we conducted an advisory patient panel from The Migraine Trust to ensure that the study addressed patient‐prioritized symptoms and that provocation procedures were acceptable and ethically appropriate.

### Study design and setting

This experimental medicine study was conducted in the National Institute for Health and Care Research King's Clinical Research Facility, King's College Hospital, London. Participants with VM, defined as described below, underwent pharmacological provocation with intravenous NTG, with repeated assessments of eye movements and symptoms obtained at baseline before provocation, after provocation, and after acute treatment with subcutaneous sumatriptan.

The original study concept involved comparison across vestibular disorders and healthy participants. This article reports the first analysis of vestibulo‐oculomotor outcomes from the parent study.

An initial pilot phase (*n* = 5) was undertaken to assess feasibility and symptom provocation. These participants were included in the final analytic cohort, and identical vestibulo‐oculomotor recording protocols were applied throughout all study phases. The pilot phase revealed greater variability in vestibular symptom responses following NTG provocation than anticipated. This challenged the assumption of a homogeneous response suitable for between subject–group comparisons (e.g., healthy participants vs. patients) and instead supported the adoption of a within‐subject repeated‐measures approach focused on individual mechanistic response profiles.

Eye movement assessments were included as prespecified secondary mechanistic outcomes in the original protocol. Before analysis of the full cohort, quantitative nystagmus burden, expressed as summed SPV across gaze positions and directions, was selected as the primary oculomotor metric for this exploratory analysis because it provided a continuous summary measure of overall nystagmus burden across gaze positions.

An a priori sample size calculation was performed for the parent (multi‐group) study to determine the number of participants required in each of the three planned study arms. No formal sample size calculation was performed specifically for the present nystagmus‐centered study; however, the present study is based on the first completed cohort (*n* = 20) before recruitment to the remaining arms was undertaken. The current sample size is actually the participants who completed study procedures at the time of exploratory analysis rather than by a power calculation targeting changes in nystagmus measures. Similar sample size has been used in studies evaluating spontaneous^5^, ^9^ and triggered^10^ attacks.

This exploratory study aimed to characterize this variability and generate preliminary estimates to inform future hypothesis‐driven designs.

Recruitment to the remaining planned study arms was not pursued after analysis of the first completed cohort demonstrated no consistent group‐level change in quantitative nystagmus measures and substantial heterogeneity in individual responses.

No data from the present analysis have been published previously.

### Participants

#### Recruitment

Participants were recruited from general neurology, headache, and neuro‐otology clinics at King's College, Charing Cross Hospital (Imperial National Health Service) and National Hospital for Neurology and Neurosurgery, Queen Square (University College London Hospitals), by the study investigators as well as through community‐based advertising, including a dedicated website, online advertisements, research volunteer recruitment circulars, and patient group advertising via the Migraine Trust, the Ménière's Society, and other relevant headache and vestibular social media platforms. Additional recruitment occurred through advertisements around the university and via local and national headache clinics.

#### Eligibility

Eligible participants were adults aged 18 to 65 years, with definite VM, diagnosed according to Bárány Society and ICHD‐3 criteria,^1^, ^3^ and no contraindications to study participation and/or NTG exposure. Use of migraine preventive therapy was permitted.

Exclusion criteria included other vestibular disorders, contraindications to NTG or triptans, pregnancy, continuous headache or vestibular symptoms, regular use of analgesics on more than 15 days per month, and use of acute analgesia within 24 h of testing. Illicit drug use and excess alcohol and tobacco consumption were also excluded, and any other medical, neurological, or psychiatric condition that, in the investigator's clinical judgment, could compromise participant safety or data interpretability.

Before performing the study protocol, all participants underwent a structured clinical assessment by a headache and neuro‐otology–trained neurologist, including comprehensive history and detailed phenotyping of spontaneous VM attacks, neurological examination, and oculo‐vestibular assessment, which included gaze holding, cover tests, spontaneous and positional nystagmus, saccadic and pursuit eye movements, as well as a horizontal and vertical head impulse test and positional maneuvers.

#### Differential diagnoses

Differential diagnoses were considered during the structured clinical interview and examination. VM was diagnosed clinically according to the joint Barany Society and ICHD‐3 criteria.^3^ The majority of participants were referred with an established diagnosis made by a headache specialist, neurologist, audiovestibular medicine physician, or ear, nose, and throat specialists, which was reconfirmed at a screening visit. Benign paroxysmal positional vertigo was assessed and ruled out using standard positional maneuvers, and Ménière's disease was excluded on clinical grounds. Neuroimaging and audiovestibular tests were performed when clinically indicated to support differential diagnosis, and 15 participants had undergone prior neuroimaging as part of routine clinical care. Other relevant cardiovascular causes of dizziness were assessed clinically, and an electrocardiogram was performed and excluded as appropriate. Neuroimaging and audiovestibular tests were performed if clinically indicated to support differential diagnosis. One participant (participant 7) is included in the tables for clinical context but excluded from all analysis; repeat review of the initial imaging identified a cerebellopontine angle abnormality that had not been reported on the original scan. One participant with a mild Chiari I malformation was included because her symptoms, typical of VM, disappeared with migraine preventive treatment.

### Experimental protocol

#### Room and positioning

All experiments were performed in a silent vestibular laboratory set up within the clinical research facility, which consisted of a room with constant ambient lighting and temperature; this ensured all variables that could interfere with autonomic and triggering conditions were kept stable and consistent across all participants and visits. Participants were seated facing the screen at a distance of 125 cm, with the center of the screen visually aligned orthogonally to Reid's plane. Minor adjustments in chin height were made to optimize eye–target alignment. Custom‐made chin and head rests were used to achieve near‐complete head stabilization during recordings.

#### NTG preparation

NTG was prepared in a 50 mL syringe at a concentration determined by participant body weight, according to a predefined dosing table. Participants were weighed on the day of infusion. Preparation and administration followed local pharmacy and clinical trial facility protocols.

#### NTG infusion and symptom monitoring

Each participant was exposed to a 0.5 μg/kg/min NTG infusion^7^, ^11^ over 20 min using an infusion pump, following 10 min in the supine position. Participants were hemodynamically monitored with noninvasive blood pressure, heart rate, and oxygen saturation measurements obtained at baseline and at 5‐min intervals throughout the infusion.

Following NTG administration, participants were interrogated for the development of headache and vestibular symptoms and systematically questioned regarding the evolution of any headache (onset, location, severity, and phenotype) and the presence of vestibular and other associated features, including canonical migraine and premonitory symptoms.

#### Sumatriptan administration

All participants received subcutaneous sumatriptan at 4 h from the beginning of the infusion to standardize assessment timing across the cohort. Rescue subcutaneous sumatriptan was offered earlier if symptoms were severe or distressing, in accordance with participant preference. Sumatriptan was selected as the acute intervention due to its well‐characterized and relatively specific mechanism of action as a serotonin 5‐hydroxytryptamine (5‐HT_1B/1D_) receptor agonist.^12^

A light breakfast was permitted on the morning of the study visit, and fasting was kept for the duration of the triggering protocol. To maintain hydration and glycemic stability, participants received intravenous 500 mL of 5% glucose in saline over the remainder of the visit. Capillary blood glucose was monitored by finger‐prick testing at baseline (prior to NTG infusion), following NTG administration, and after subcutaneous sumatriptan.

### Eye movement evaluation

#### Eye movement recording

Eye movement recordings during the NTG condition were performed at the individual peak of vestibular symptom intensity if symptoms were reported, or at 3 h from infusion onset in participants who did not develop vestibular symptoms, to standardize timing across participants while maximizing the likelihood of capturing symptom‐related oculomotor changes.

Data were collected from both eyes at three time points: baseline (pre‐NTG), following NTG administration, and after sumatriptan administration. Video‐oculography was performed with a 3D binocular eye‐tracker device (Interacoustics VisualEyes 525). The head‐mounted unit incorporates two infrared cameras and communicates with the VisualEyes (Beta version 103.2.0.2948) software for image processing. Eye image acquisition was performed at 100 fps (capture resolution per camera: 320 × 240 pixels), with an eye movement resolution of 0.3° and a dynamic range of ±45° horizontally and ±25° vertically. Infrared illumination was provided by a 950‐nm IR LED (1.5 mW/cm^2^). Prior to calibration, the eye images were centered/aligned using the system's centering function (and manual adjustment as needed), after which video‐nystagmography calibration was performed using the automated 5‐point calibration procedure within the VisualEyes software. Head‐motion sensor Micromedical VORTEQ 2nd Generation was used for the relevant tests, for example, positional testing.

Visual stimuli were presented on a secondary ultrahigh definiton 4K screen, positioned in front of the participant and controlled via the acquisition software. Refresh rate was 60 Hz, and hardware delay was adjusted to be close to 0 ms. The stimulus consisted of a central target presented on a uniform background. Visual stimuli were presented on a calibrated 4K display under controlled conditions. Stimulus parameters (position, duration, and timing described below; size 1; target color green; background black) were standardized and kept constant across participants and recording sessions.

#### Nystagmus analysis

Nystagmus was analyzed offline using VisualEyes software with manual verification against the raw video recordings. Nystagmus was considered present if more than two consecutive beats were observed. Data were visually inspected and filtered; artifacts were removed prior to analysis. Participants were instructed to fixate on the target for 20 s. After this period, visual input was occluded by covering the eyes in complete darkness, and participants were asked to maintain their gaze toward the remembered target location as if it were still present. Quantification was performed in the absence of visual fixation (eyes covered and complete room darkness). Slow‐phase velocity (°/s) was calculated as the peak velocity observed after occlusion, within the 20 to 40 s interval of the recording. Each gaze condition had a total duration of 40 s.

Gaze‐evoked nystagmus was assessed in five gaze positions (primary position and 20° to the left and right, and 11° up and down). The presence of nystagmus and corresponding SPV in each gaze position and direction were recorded. To derive a single measurement per NTG condition, we summed absolute (i.e., unsigned) SPV measurements across standardized gaze positions and directions. This aggregation approach is analogous to established vestibular composites such as the “total caloric response/total SPV,” designed as the sum of peak SPV across repeated stimuli,^13^, ^14^ and in this study would simplify within‐subject comparisons and statistics (e.g., a patient with a baseline right‐beating nystagmus of 1°/s who after NTG increases to 2°/s but also adds an upbeating nystagmus of 3°/s would be quantified as a total change of SPV of 4°/s). All traces were visually inspected to rule out the unlikely possibility that a participant could have had a baseline nystagmus disappear after NTG but a different nystagmus of a similar SPV appear (e.g., a patient with a baseline 4°/s right‐beating nystagmus that disappears after NTG but is replaced by 4°/s downbeating nystagmus would have been quantified as no NTG‐induced change in SPV).

### Symptom assessment

Headache intensity and vestibular symptoms were assessed using ordinal numeric rating scales (0 to 10, maximum described as “the most intense headache ever imagined” or “vestibular/dizzy symptoms so intense that you would collapse”). Participants were encouraged to report the presence of associated perceptual symptoms, including visual aura, derealization/depersonalization, and visual distortions (metamorphopsia), during the provoked migraine state. Participants presenting visual symptoms were asked to indicate their perceived motion by manipulating a simple schematic representation of their own body (a small avatar of self‐position in space), adjusting it to match their sensation of movement.

### Statistical analysis

Descriptive statistics were used to summarize baseline characteristics and feasibility data and are reported as mean (standard deviation) or median (interquartile range [IQR]) as appropriate. The Shapiro–Wilk test and visual inspection of histograms were used to evaluate data distribution. Categorical data are reported as counts and percentages.

Gaze‐evoked nystagmus SPV values demonstrated substantial right skewness and a high proportion of zero values. To account for these distributional properties and repeated within‐subject measurements, generalized linear mixed‐effects models were fitted using the glmmTMB package in R version 4.4.2 (R Foundation for Statistical Computing, Vienna, Austria) in the RStudio 2024.12.0 Build 467 integrated development environment (Posit PBC, Boston, MA, USA). A Tweedie mixed‐effects model with log link was used as the primary analysis to evaluate overall nystagmus burden across conditions (baseline, NTG, and sumatriptan), with condition included as a fixed effect and participant as a random intercept. As an exploratory sensitivity analysis, a two‐part hurdle approach was additionally applied, consisting of a logistic mixed‐effects model that takes into account presence versus absence of nystagmus, followed by a Gamma mixed‐effects model assessing nystagmus severity (SPV) among positive observations only. Statistical testing was two‐tailed and considered significant at a *p* < 0.05.

## RESULTS

### Participant flow

A total of 209 individuals were screened for eligibility. Of these, 188 were not included because they were healthy volunteers, had alternative vestibular diagnosis, did not fulfill VM diagnostic criteria, or met one or more exclusion criteria. Twenty‐one participants with definite VM were enrolled and completed the study protocol, including five participants recruited during the initial pilot phase. Following post hoc review of neuroimaging, one participant (participant 7) was excluded from all analyses because of a previously unreported cerebellopontine angle abnormality. The final analytic cohort therefore comprised 20 participants (Figure 1).

**FIGURE 1:**
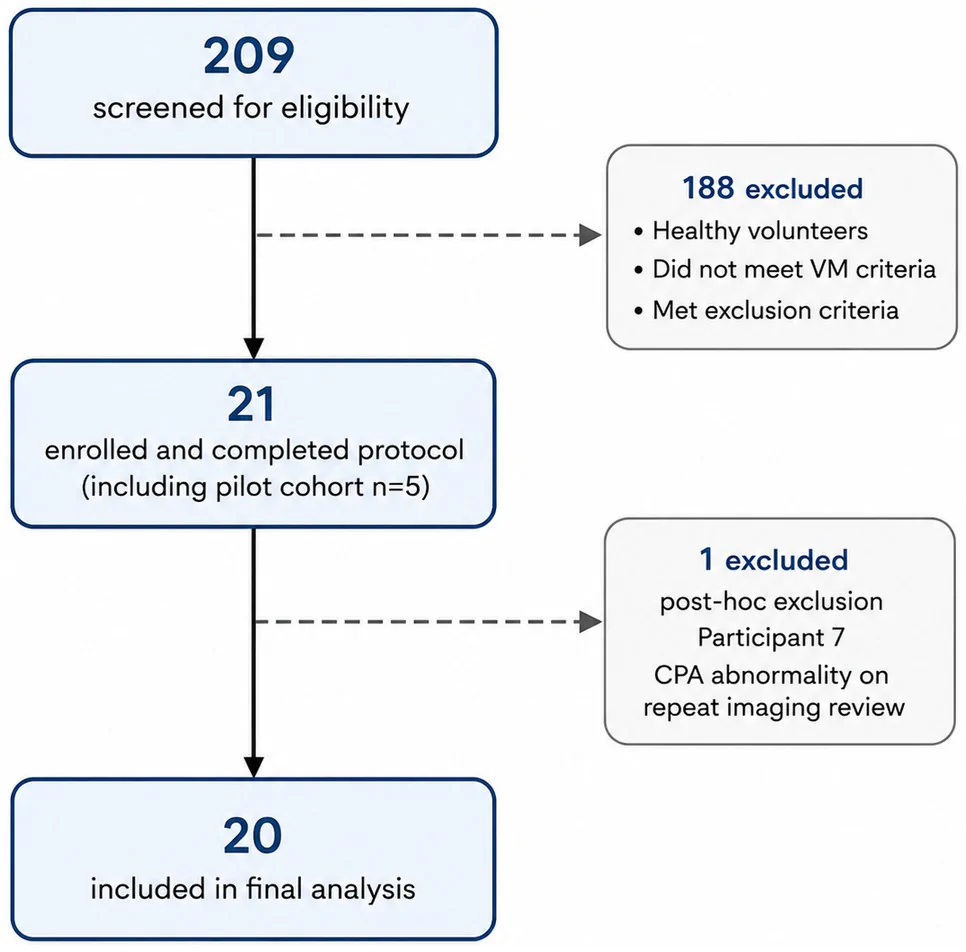
Participant flowchart. [Color figure can be viewed at wileyonlinelibrary.com]

### Demographics and headache phenotype

Seventeen of 21 participants were female. Ethnicity was self‐reported by participants. The cohort was predominantly white British, with additional participants identifying as Indian, Central European, Mexican, South American, Arab, and Scandinavian. Age was slightly skewed, with a median (IQR) 32 (29 to 38) years. Migraine‐related disability varied widely (Table 1), with MIDAS^15^ scores ranging from minimal to very severe, whereas Dizziness Handicap Inventory scores indicated predominantly mild‐to‐moderate vestibular handicap, with a smaller proportion in the severe range. Aura was common, particularly visual and somatosensory types, with some participants reporting more complex features including dysphasic aura. Comorbidities were frequent and heterogeneous, including functional disorders, connective tissue traits, and systemic conditions. A lifetime history of head trauma severe enough to seek assistance was reported in 45% of participants, most commonly related to falls or motor vehicle collisions (Table 1). On NTG, all participants developed a headache that worsened with movement. Assessment of fulfillment of the ICHD‐3 duration criterion for migraine was not possible because all participants received acute treatment with sumatriptan within 4 h from the start of the infusion, potentially aborting the attack before the minimum required duration.

**TABLE 1:** Demographics.

Participant	Sex	Age	MIDAS	DHI	Aura	Comorbidities	Significant head trauma
1	F	33	17	52	Visual	Asthma, endometriosis	Fall down stairs at age 20 years, associated with retrograde amnesia
2	M	31	39	62	Visual, somatosensory	Asthma, IBS, eczema	No
3	F	27	15	52	Visual	Endometriosis, IBS, PCOS, hypermobility, TMJD, septoplasty	Frontal head trauma with loss of consciousness
4	M	33	10	48	No	No	No
5	F	52	63	58	Visual	Chiari 1 R>L, Ehlers‐Danlos syndrome, asthma, IBS, osteoarthritis	Interpersonal physical assault involving head impact
6	M	42	15	30	Visual	IBS, HIV, Bell's palsy	No
*7 (excluded)*	*F*	*42*	*4*	*30*	*Visual*	*Arachnoid cyst inferior aspect of right cerebello‐pontine reflux, eczema*	*Early childhood accidental drop (age 2 years), resulting in head impact*
8	F	49	0	10	No	IBS	No
9	F	37	46	30	Visual, somatosensory, dysphasic	SUNCT (currently inactive), hypermobile and costochondritis	Sports‐related head impact (football) at age 20 years
10	F	27	43	84	Visual, somatosensory	Graves' disease, IBS, joints pain under study and recurrent hives	No
11	F	24	5	52	No	Hypermobile, previous Miller Fisher syndrome, small nonsecretory prolactinoma	No
12	M	32	40	30	Visual	Reflux, hiatus hernia	Motor vehicle collision with whiplash injury at age 27 years
13	F	32	25	48	Somatosensory	IBS, eczema	Fall‐related head injury with occipital impact
14	F	29	115	60	Somatosensory	Asthma, IBS, insomnia	Motor vehicle collision with whiplash injury at age 19 years
15	F	45	15	30	Visual	IBS, acne, insomnia	Fall on ice resulting in head impact
16	F	23	29	58	No	No	Motor vehicle collision with whiplash
17	F	33	39	50	Somatosensory	No	No
18	F	30	39	28	Somatosensory	Hypermobility, facial palsy, sympathectomy	No
19	F	30	38	48	Visual, somatosensory	Asthma, IBS, hyperactive bladder, hypermobility, joint pain, PTSD	No
20	F	23	13	18	Visual, dysphasic	Asthma, chest pain, insomnia	No
21	F	49	123	28	No	Hypothyroidism, rheumatoid arthritis, anemia, medium frequencies hypoacusis	No

*Note*: Demographic and clinical characteristics of the study cohort. Aura types include visual, somatosensory and dysphasic, multiple aura types may be present in the same participant. Comorbidities were self‐reported. Significant head trauma refers to a history of head injury with potential clinical relevance. Participant 7 (in italics) was excluded from all statistical analyses after review of neuroimaging identified a previously unreported cerebellopontine angle abnormality that may have represented an alternative cause of vestibular symptoms. The participant is retained in descriptive tables for transparency and clinical context.

Abbreviations: DHI, Dizziness Handicap Inventory; F, female; HIV, human immunodeficiency virus; IBS, irritable bowel syndrome; L, left; M, male; PCOS, polycystic ovary syndrome; PTSD, post‐traumatic stress disorder; R, right; SUNCT, short‐lasting unilateral neuralgiform headache attacks with conjunctival injection and tearing; TMJD, temporomandibular joint disorder.

Maximum headache intensity during NTG‐provoked migraine was skewed and bimodal; values are therefore reported as median (IQR), 6 (4 to 7). Headache latency from infusion onset showed a right‐skewed distribution, with a median of 8.5 (4.3 to 23.8) min.

Headache quality was predominantly pressure‐like (45%) or throbbing (40%), with mixed phenotypes in 15%. Pain most frequently involved fronto‐orbital regions, often affecting multiple cranial territories (Table 2). Although some participants had an early headache followed by later worsening after NTG administration, this biphasic pattern was less distinct than previously reported, with many individuals experiencing persistent headache throughout the observation period. No participant required earlier rescue treatment.

**TABLE 2:** Headache responses.

Participant	Intensity	Onset	Quality	Location	AIWS	Visuals
1	4	5 min	UNK	Frontal	NA	NA
2	2	10 min	Throbbing	Frontal	Altered time perception	NA
3	7	120 min	Pressure	Bilateral frontal	NA	NA
4	1	50 min	Pressure and throbbing	Bilateral temporal	NA	NA
5	4	5 min	Pressure	Left frontal, periorbital and parietal	Derealization (detached from reality)	Left hemianopsia, diagonal lines such as rain, rainbow pattern and ianothinopsia (purple vision)
6	8	35 min	Throbbing	Bilateral fronto‐parietal–temporal	Depersonalization	Photopsias, erythropsia (red vision) and chloropsia (green vision)
*7 (excluded)*	*7*	*3 min*	*Throbbing*	*Left periorbital*	*NA*	*NA*
8	7	5 min and 110 min	Pressure	Bifrontal and fronto‐parietal	NA	NA
9	4	2 min	Throbbing	Left temporo‐parietal and right frontal	NA	Afterimages and chloropsia (green vision) during OKN
10	8	15 min	Pressure	Parietal and periorbital	Derealization (dreamy sensation)	Floaters and halos
11	6	1 min	Throbbing	Left occipital and periorbital	NA	NA
12	4	150 min	Throbbing	Pressure in ear and inion + occipital (throbbing)		Left eye scotoma, afterimages
13	7	9 min	Pressure	Left retro‐orbital	NA	NA
14	7	8 min	Throbbing	Bifrontal, temporal, vertex	NA	Stars pattern bilateral
15	8	60 min	Pressure	Bioccipital	NA	NA
16	6	1 min	Throbbing	Left retro‐orbital	NA	NA
17	6	2 min	Pressure	Right frontal	NA	Photopsias
18	4	9 min	Pressure	Holocranial	NA	NA
19	4	5 min	Pressure	Bifrontal	Derealization (surroundings look unreal), macropsia, dysmorphopsia	Scintillating scotoma‐R, phosphene‐L
20	3	20 min	Pressure	Right supraorbital	Metamorphopsia (tiles dispersing or slow boiling)	Scotomas (3 bilateral)
21	8	2 min	Throbbing	Occipital and frontal	NA	Scotomas and floaters

*Note*: Characteristics of headache and associated perceptual symptoms following NTG provocation. Intensity refers to headache severity on a 0 to 10 numerical rating scale. Onset indicates time from NTG administration to symptom onset. Quality and location were self‐reported. AIWS included derealization, depersonalization, macropsia, micropsia, and other perceptual distortions. Visual symptoms include photopsias, scotomas, afterimages, color distortions, and visual field abnormalities. Participant 7 (in italics) was excluded from all statistical analyses after retrospective review of neuroimaging identified a previously unreported cerebellopontine angle abnormality that may have represented an alternative cause of vestibular symptoms. The participant is retained in descriptive tables for transparency and clinical context.

Abbreviations: AIWS, Alice in Wonderland syndrome; NA, not reported; NTG, nitroglycerin; UNK, unknown.

### Vestibular symptoms

Vestibular symptoms were triggered in 14/20 participants following NTG, with a median (IQR) maximum intensity of 2.5 (0 to 4) in the total cohort and 3 (2.25 to 4.75) in those who developed vestibular symptoms. Vestibular symptom latency was highly variable, with a median (IQR) of 36.5 (10.3 to 107.5) min.

Vestibular symptoms showed marked heterogeneity across participants, encompassing classical vestibular symptoms that were mostly described using plain‐language metaphors or comparisons chosen *motu proprio* by the participant. These included rotational vertigo (e.g., roll spinning, diagonal spinning, yaw spinning), body oscillatory sensations (e.g., medio‐lateral swaying, vertical bobbing), and translational illusions of self‐motion (e.g., floating in space, falling sensations, forward deceleration as in a bus or train stopping).

Intensity of vestibular symptoms ranged from mild to moderate intensity, supporting a nonuniform triggering pattern between individuals. We note that participant 7 developed both headache and vertigo following NTG administration. Although this observation was considered clinically interesting, the presence of the structural abnormality precludes attribution of these symptoms specifically to VM mechanisms. We have therefore limited reporting of this case to descriptive context and have not included the data in any statistical analyses.

### Spontaneous (primary position) and gaze‐evoked nystagmus

Total spontaneous and gaze‐evoked nystagmus burden, expressed as the summed SPV (°/s) across gaze positions and directions, showed substantial interindividual variability and a high proportion of zero values across conditions (i.e. no nystagmus). Median (IQR) summed SPV was 2 (1 to 3.25) at baseline, 1.5 (0 to 4.75) following NTG, and 1 (0 to 2.75) after sumatriptan. In the primary Tweedie generalized linear mixed‐effects model, condition was not significantly associated with total nystagmus burden. Compared with baseline, neither NTG (*β* = 0.118, SE = 0.267, *p* = 0.658) nor sumatriptan (*β* = −0.078, SE = 0.278, *p* = 0.780) showed a significant effect. Estimated marginal means were 1.87 (95% confidence interval [CI] 1.02 to 3.41) at baseline, 2.10 (95% CI 1.16 to 3.80) following NTG, and 1.73 (95% CI 0.93 to 3.19) after sumatriptan. Tukey‐adjusted post hoc pairwise comparisons were also nonsignificant. Pairwise comparisons demonstrated no significant differences between conditions after Tukey correction (all *p* > 0.75) (Figure 2). Exploratory hurdle modeling likewise demonstrated no significant effect of condition on either the probability of exhibiting any nystagmus or the nystagmus severity among positive observations (Figure 3). Importantly, no participant developed a marked or unequivocally pathological nystagmus or oculomotor abnormality following NTG. There were no missing data.

**FIGURE 2:**
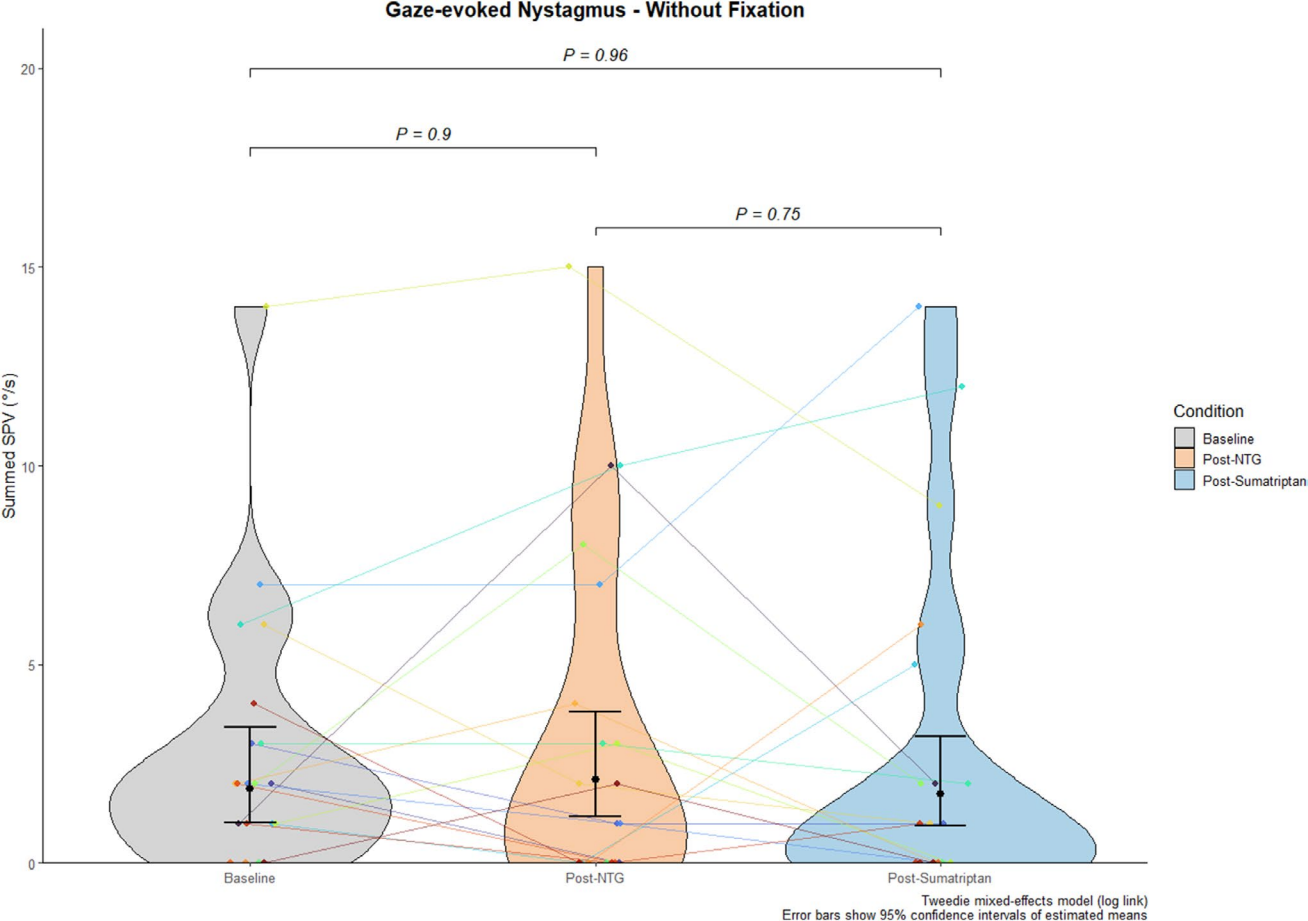
Distribution of summed gaze‐evoked nystagmus burden without fixation across baseline, after NTG, and post‐sumatriptan conditions (*n* = 20). Total nystagmus burden was expressed as the summed SPV (°/s) across gaze positions and directions. Violin plots illustrate the distribution of raw data, whereas individual points represent participant‐level observations. Dots and error bars indicate model‐estimated marginal means and 95% confidence intervals derived from Tweedie generalized linear mixed‐effects models, with participant included as a random intercept and condition as a fixed effect. *p* values shown represent Tukey‐adjusted pairwise comparisons between conditions. No significant between‐condition differences in total nystagmus burden were observed. NTG, nitroglycerin; SPV, slow‐phase velocity. [Color figure can be viewed at wileyonlinelibrary.com]

**FIGURE 3:**
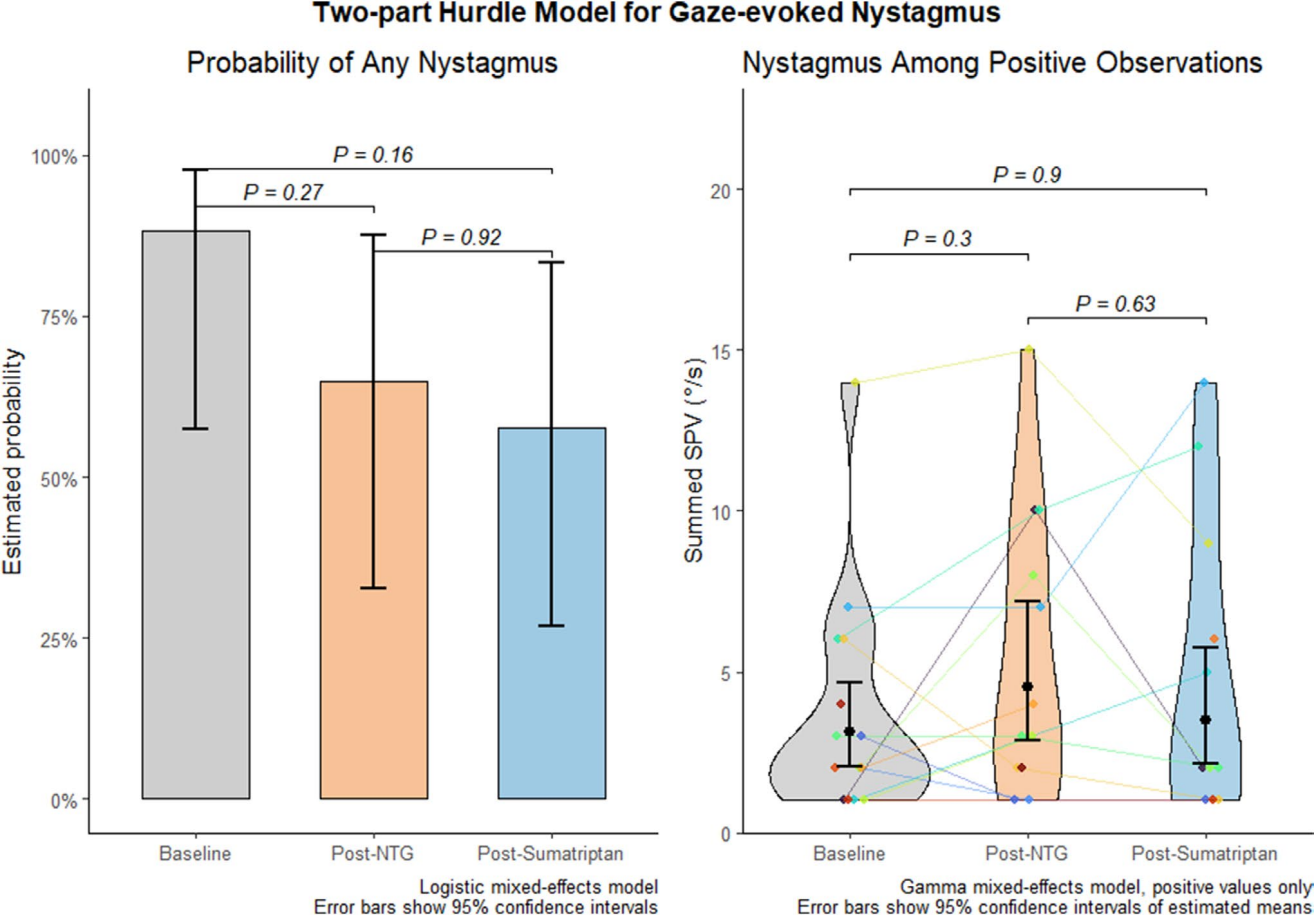
Two‐part hurdle modeling of gaze‐evoked nystagmus without fixation across baseline, after NTG, and post‐sumatriptan conditions (*n* = 20). Left panel: Logistic mixed‐effects model estimating the probability of exhibiting any nystagmus across conditions. Bars and error bars represent model‐estimated probabilities and 95% confidence intervals, respectively. Right panel: gamma mixed‐effects model evaluating nystagmus severity among positive observations only. Violin plots illustrate the distribution of summed SPV (°/s) values, whereas individual points represent participant‐level observations. Dots and error bars indicate estimated marginal means and 95% confidence intervals. *p* values shown represent Tukey‐adjusted post hoc pairwise comparisons estimated from the hurdle models. No significant between‐condition differences were observed for either the probability or severity of nystagmus. NTG, nitroglycerin‐induced migraine; SPV, slow‐phase velocity. [Color figure can be viewed at wileyonlinelibrary.com]

In a subset of participants, vestibular symptoms appeared temporally associated with ocular movements recorded by video‐oculography (Table 3), although this observation was not formally quantified and should be interpreted cautiously.

**TABLE 3:** Vestibular subjective responses and VNG correlations.

Participant	D1 Intensity	D1 Onset	D1 Description	D2 Intensity	D2 Onset	D2 Description	VNG correlations	Similarity to spontaneous attacks
1	6	180 min	Spinning roll	0	NA	NA	“Earthquake” sensation coinciding with high‐amplitude corrective saccadic movement of right drift (during gaze center, without fixation)	100%
2	4	30 min	Head‐eye visual lag	5	90 min	NA	Bilateral reduction of in v‐HIT gain (average 11%)	90%
3	3	120 min	Motion sickness	0	NA	NA	No	100%
4	0	NA	NA	0	NA	NA	No	NA
5	3	180 min	Train stopped suddenly	0	NA	NA	No	UNK
6	5	21 min	Spinning diagonal	0	NA	NA	No	UNK
*7 (excluded)*	*4*	*180 min*	*Falling*	*0*		*Car sickness and lightheadedness during BP drop after NTG*	*Rollercoaster sensation (tilt‐table backward) during head straight back (coinciding with UBN, when UBN disappeared, the sensation disappeared, despite still lateral and torsional nystagmus). Mild UBN evident also with fixation*	*UNK*
8	0	NA	NA	0	NA	NA	NA	NA
9	1	60 min	Unsteadiness	3	190 min	Pitch forward and left (diagonal) upon sitting from Hallpike	During Left Hallpike without fixation (initial left upbeat nystagmus, 3 beats)	UNK
10	5	43 min	Floating (decubitus) Swaying (sitting)	0	NA	NA	UBN visible with ophthalmoscope. Good correlation horizontal and vertical nystagmus with her perception of movement in video	UNK
11	0	NA	NA	0	NA	NA	NA	NA
12	2	11 min	Bobbing and spinning diagonal		19 min	Bobbing	Afterimages move left and right (LBN that increases with the increase in SPV and also UBN). During positional maneuvers, his perception of movement correlates with these eye movements	60%
13	0	NA	NA	0	NA	NA	NA	NA
14	1	10 min	Teacup spinning	0	NA	NA	Right‐spinning “teacup,” sensation which coincides with LBN 2°/s and modulates with SPV	10%
15	5	5 min	Floating	4	34 min	Not processing head/eyes movements	UBN while supine coincides with floating sensation. During baseline visit had DBN instead.	100%
16	0	NA	NA	0	NA	NA	NA	NA
17	1	70 min	Bed bobbing and swaying	1	NA	NA	Moves hand‐held avatar similarly to her DBN when sitting on the couch	100%
18	0	NA	NA	0	NA	NA	NA	NA
19	4	7 min	Swaying	3	160 min	Spinning CCW	No	UNK
20	3	190 min	NA	0	NA	NA	NA	UNK
21	3	5 min	Jerky tilts to the left	3	5 min	Unsteadiness and self‐motion	Jerk sensations coincide with the frequency of UBN. Still has SWJ but not upbeats when the dizziness disappeared	UNK

*Note*: Vestibular responses following NTG provocation. Intensity refers to subjective dizziness severity on a 0 to 10 numerical rating scale. Onset indicates time from NTG administration to symptom onset. Descriptions of dizziness were self‐reported. VNG correlations refer to qualitative associations between reported symptoms and findings on VNG or clinical examination. Similarity to spontaneous attacks reflects participants' subjective comparison with their typical migraine‐related vestibular symptoms. Participant 7's responses may also be of interest to readers in illustrating responses in individuals with potential secondary causes of dizziness. Participant 7 was excluded from all statistical analyses after retrospective review of neuroimaging identified a previously unreported cerebellopontine angle abnormality that may have represented an alternative cause of vestibular symptoms. The participant is retained in descriptive tables for transparency and clinical context.

Abbreviations: BP, blood pressure; CCW, counterclockwise; D1, D2, dizziness types 1 and 2; DBN, downbeat nystagmus; LBN, left‐beating nystagmus; NA, not reported; NTG, nitroglycerin; SPV, slow‐phase velocity; SWJ, square wave jerks; UBN, upbeat nystagmus; UNK, unknown; v‐HIT, video head impulse test; VNG, video‐nystagmography.

### Visual and other perceptual symptoms

Visual perceptual symptoms were present in 10/20 of episodes, and Alice in Wonderland syndrome^16^ (AIWS)‐like or dissociative phenomena in 6/20. These included depersonalization, described as “legs are disconnected from the body” (participant 6), and derealization (3/20), reported as sensations of “being in a dream” (participant 10), “being detached from reality” (participant 5), or “the surroundings are unreal” (participant 19). In the latter participant, the symptoms evolved into macropsia and pelopsia (“objects look bigger and distances look closer”), and dysmorphopsia, reported as “everything looks like modern art.” Participant 20 described metamorphopsia, stating that “the floor seems to be boiling and the tiles' patterns are dispersing.” Participant 2 described altered time perception, whereby everything around him appeared to be in slow motion despite an unchanged sense of subjective time.

### Response to sumatriptan

Although the study was not designed to formally evaluate treatment response, all participants were monitored until symptom severity had improved to a mild level (≤2 on the study rating scale) following sumatriptan administration, and no participant was discharged with ongoing moderate or severe symptoms.

## DISCUSSION

In this exploratory study, intravenous NTG reliably triggered migraine headache in all participants and provoked vestibular symptoms in a majority of individuals with VM (14/20). However, using quantitative assessment of eye movements, NTG did not produce consistent group‐level changes in gaze‐evoked nystagmus, total SPV, or induced robust vestibulo‐ocular abnormalities. The apparent dissociation between symptom provocation and the absence of a detectable uniform oculomotor signature suggests that any NTG‐related vestibulo‐oculomotor changes may be subtle, heterogeneous, temporally variable, or insufficiently captured by the current experimental paradigm. Given the modest sample size, substantial interindividual variability, composite outcome measures, and nonuniform recording time points—with symptoms recorded at individual peaks versus asymptomatic participants recorded at a fixed 3‐h mark—these findings should not be interpreted as evidence for the absence of vestibulo‐oculomotor alterations in VM but rather as an inability to detect a consistent group‐level effect under the present study conditions.

Notably, the intensity of vestibular symptoms was generally mild to moderate across the cohort. This contrasts with the robust and universal triggering of headache and may suggest that the threshold for inducing vestibular symptoms in VM is higher than that for headache.^17^ A similar dissociation has been described in NTG‐provoked migraine, for which headache is more consistently elicited than associated features such as nausea, photophobia, or phonophobia.^7^, ^11^, ^17^ This raises the possibility that NTG may engage core migraine headache‐generating pathways more readily than those mediating migraine dizziness‐generating pathways, or that additional network recruitment is required to generate clinically significant dizziness.

A central finding of this study is the marked interindividual heterogeneity in the phenomenology, intensity, and temporal profile of provoked vestibular symptoms. Participants described qualitatively distinct perceptions of self‐motion, encompassing rotational, oscillatory, and translational illusions, often using simple comparisons and metaphors, such as “I feel swaying,” “I feel like inside a fairground teacup,” or “the chair is bobbing up and down.” The wide dispersion in vestibular symptom latency and the nonuniform intensity profile further suggests that NTG does not act on a single vestibular pathway in VM but rather engages multiple nodes within distributed vestibular‐visual‐somatosensory networks. An alternative but not mutually exclusive interpretation is that the finding indicates that the symptom phenotype of VM is highly variable in contrast to the ubiquitous strong rotational vertigo observed in peripheral vestibular disorders such as vestibular neuritis or Meniere's disease.

In contrast to headache, which typically developed earlier following NTG infusion, vestibular symptoms showed a broader temporal distribution. Although participants were monitored throughout the experimental session and contacted 24 h after, delayed vestibular symptoms beyond this time window cannot be fully excluded and warrant consideration in future studies with extended follow‐up; however, no patient contacted the research team to report a delayed vertigo episode.

The absence of a consistent change in nystagmus across conditions argues against a uniform brainstem or peripheral vestibular driver of NTG‐induced vestibular symptoms in VM. Instead, these findings are more compatible with a distributed model in which NTG modulates central sensory integration processes in a state‐dependent and individually variable manner. Other paradigms may be more sensitive to state‐dependent changes. Head‐shaking nystagmus, although mechanistically informative, may itself provoke migraine or vestibular symptoms and therefore poses interpretive challenges in provocation paradigms.

The frequent co‐occurrence of vestibular symptoms with complex visual and perceptual phenomena (including Alice in Wonderland‐like features such as metamorphopsia or derealization and dissociative symptoms) further supports predominant involvement of higher‐order multisensory cortical regions, particularly parietal and temporo‐occipital areas implicated in self‐motion perception and vestibulo‐visual integration. This aligns with contemporary models of VM as a disorder of altered multisensory inference rather than a purely peripheral or brainstem vestibular pathology, exemplified by the poor spatial orientation observed in these patients when compared to structural vestibular disorders.^18^ Conditions such as visual snow syndrome, tinnitus, AIWS, and persistent postural–perceptual dizziness (PPPD) are all highly comorbid with migraine and share a phenotype characterized by heightened sensitivity to both external and internal physiological stimuli, reflecting abnormalities in multimodal sensory integration and sensory gating.^19^, ^20^ A unifying pathophysiological framework proposes that these conditions may represent network‐level disorders involving an imbalance between excitatory and inhibitory signaling, leading to hyperexcitability or disinhibition within primary and higher‐order perceptual cortices.^21^ Whereas such mechanisms have been particularly described in visual snow syndrome and related conditions,^22^, ^23^ similar network dysfunction has also been implicated in VM. Indeed, prior work combining vestibular and oculomotor measures supports the presence of abnormal visuo‐vestibular interactions and altered sensory thresholds in VM.^24^ Notably, these alterations may not be uniform across contexts because vestibular thresholds may differ between spontaneous and experimentally induced (e.g., NTG‐triggered) attacks, suggesting dynamic state‐dependent modulation of sensory processing. In addition to sensory networks, dysfunction may extend to attentional mechanisms, particularly the salience network, and emotional regulation, including limbic circuits, thereby contributing to altered perceptual salience, symptom persistence, and increased symptom burden.

This potential coupling raises the possibility that, in some individuals, oculomotor signals or altered central processing of eye movement‐related afferents may contribute to symptom perception, whereby internally generated movement signals are misinterpreted as externally driven self‐motion. However, without a control group, it remains unclear whether such associations are specific to VM or may also occur in healthy individuals under similar experimental conditions. The apparent restriction of this phenomenon to a subset of participants further underscores the mechanistic heterogeneity of VM.

Several methodological considerations are relevant. Preventive treatments were permitted and may have attenuated responses to NTG in some individuals. In addition, exclusion of participants with continuous symptoms enabled clearer attribution of changes to NTG but may have biased the sample toward milder or more intermittent phenotypes.

The study was not specifically powered to detect small differences in nystagmus burden between conditions. Nevertheless, the absence of any consistent directional effect across the primary Tweedie model, hurdle analyses, estimated marginal means, and individual participant observations, together with the lack of any clinically apparent pathological nystagmus following NTG administration, suggest that under the conditions studied and within this cohort, any effect is unlikely to be large.

It is also challenging to reconcile our findings with previous reports in the literature describing abnormal eye movements during the ictal or even post‐ictal phases of VM episodes. Several studies have documented oculomotor abnormalities, including gaze‐evoked and positional nystagmus, during spontaneous attacks,^5^, ^25^ although these findings were variable, often of low amplitude, and also frequently observed in the absence of fixation. These findings are in line with recent experimental work in episodic migraine,^10^ which also reported oculomotor responses following sublingual NTG in a small number of participants.

No definite explanation for such discrepancy can be offered, but a number of factors may account for these differences. First, the intensity of vestibular‐type symptoms elicited in our study was generally mild across participants. This raises the possibility that the threshold for inducing dizziness or vestibular symptoms may be higher than that required to trigger headache in migraine; this has been suggested in prior NTG‐provocation studies in which associated migrainous features (e.g., nausea, photophobia) are not consistently reproduced. In keeping with this, pathological nystagmus may emerge only at higher levels of vertigo, consistent with the relatively mild dizziness observed in our cohort. Second, the temporal profile of vestibular symptom development may differ from that of headache. Although assessments were performed within the expected peak window following NTG administration, it is possible that more prominent vestibular features, including nystagmus, may emerge later, beyond the 4‐h observation period used in this study. An additional consideration is the frequent overlap between VM and other vestibular disorders^26^ that may themselves produce spontaneous or positional nystagmus, including benign paroxysmal positional vertigo,^27^ Ménière's disease,^28^ and episodic ataxias.^29^ Although it is not possible to determine the contribution of such factors to individual studies, variation in the prevalence of comorbid vestibular disorders, together with differences in methodology and timing of assessment, may partly contribute to the heterogeneity of oculomotor findings reported in the VM literature.^5^, ^9^, ^10^, ^25^, ^30^, ^31^, ^32^ The predominance of subjective vestibular and perceptual symptoms in the absence of consistent objective vestibulo‐ocular abnormalities may also have implications for the broader spectrum of functional dizziness disorders. In particular, milder VM presentations may share clinical features with PPPD, including altered self‐motion perception, visual dependence, and sensory hypersensitivity, potentially contributing to overlap between these diagnostic constructs in clinical practice.

Future studies should investigate how other migraine‐associated symptoms, such as nausea, photophobia, and phonophobia, as well as premonitory symptoms, interact with vestibular symptoms and complex visual experiences; this may help further characterize the multidimensional phenotype of NTG‐triggered VM attacks.

## CONCLUSION

Our study shows that NTG provocation can evoke VM symptoms without eliciting a consistent vestibulo‐oculomotor pattern at the group level. Instead, responses were heterogeneous, with variability in symptom profile and no uniform oculomotor changes, and eye movement patterns corresponding closely to participants' reported perceptual experiences. Future mechanistic studies in VM may therefore benefit from stratifying participants by dominant perceptual phenotype or network signature, and from incorporating multimodal markers of cortical network function (e.g., functional imaging, perceptual tasks probing sensory integration, or computational modeling of multisensory inference) rather than relying solely on oculomotor outputs as primary endpoints.

## AUTHOR CONTRIBUTIONS

**Maria Dolores Villar‐Martinez:** Conceptualization; investigation; writing – original draft; methodology; visualization; writing – review and editing; software; formal analysis; project administration; data curation; validation. **Francesca Puledda:** Conceptualization; investigation; writing – original draft; validation; visualization; writing – review and editing; supervision. **Caroline Domingos‐Belo:** Writing – original draft; investigation; data curation. **Adolfo M. Bronstein:** Conceptualization; investigation; writing – original draft; writing – review and editing; visualization; validation; methodology; supervision; formal analysis. **Peter J. Goadsby:** Conceptualization; investigation; funding acquisition; writing – original draft; methodology; validation; visualization; writing – review and editing; software; supervision; resources; project administration.

## CONFLICT OF INTEREST STATEMENT

**Caroline Domingos‐Belo** and **Maria Dolores Villar‐Martinez** do not have any conflicts of interest relevant to this manuscript. **Francesca Puledda** has received consulting and speaker honoraria from TEVA, Abbvie, Organon, Lundbeck, and Pfizer. **Adolfo M. Bronstein** reports having received honoraria for publications and editorial work from Brain Sciences, Cambridge University Press, and Oxford University Press in the last 36 months but not related to the work reported here. **Peter J. Goadsby** reports, over the last 36 months, unrelated to the work described, personal fees for consulting from Abbvie, CoolTech, Dr. Reddy's, Epalex, Ipsen, Kallyope, Linpharma, Lundbeck, Orion Pharma, Pfizer, PureTech Health, Satsuma, Scilex Pharmaceuticals, Seaport Therapeutics, Septurna Pharmaceuticals, and Teva Pharmaceuticals; personal fees for advice through Gerson Lehrman Group, Guidepoint, and SAI Med Partners; fees for educational materials from CME Outfitters and WebMD; and publishing royalties or fees from Massachusetts Medical Society, Oxford University Press, UptoDate, and Wolters Kluwer.
